# [X]uniqMAP: unique gene sequence regions in the human and mouse genomes

**DOI:** 10.1186/1471-2164-7-249

**Published:** 2006-10-06

**Authors:** José L Jiménez, Richard Durbin

**Affiliations:** 1 Wellcome Trust Sanger Institute, Genome Campus, Hinxton, Cambridge CB10 1SA, UK

## Abstract

**Background:**

Current approaches for genome-wise functional analyses, such as microarray and RNA interference studies, rely on the specificity of oligonucleotide sequences to selectively target cellular transcripts. The design of specific oligos involves the determination of unique DNA regions in the gene/transcripts of interest from the targeted organism. This process is tedious, time consuming and it does not scale up for high-throughput studies.

**Description:**

Taking advantage of the availability of complete genome sequence information for mouse and human, the most widely used systems for the study of mammalian genetics, we have built a database, [X]uniqMAP, that stores the precalculated unique regions for all transcripts of these two organisms. For each gene, the database discriminates between those unique regions that are shared by all transcripts and those exclusive to single transcripts. In addition, it also provides those unique regions that are shared between orthologous genes from the two organisms. The database is updated regularly to reflect changes in genome assemblies and gene builds.

**Conclusion:**

Over 85% of genes have unique regions at least 19 bases long, with the majority being unique over 60% of their lengths. 14482 human genes share exactly at least a unique region with mouse genes, though such regions are typically under 40 bases long. The full data are publicly accessible online both interactively and for download. They should facilitate (i) the design of probes, primers and siRNAs for both small- and large-scale projects; and (ii) the identification of regions for the design of oligos that could be re-used to target equivalent gene/transcripts from human and mouse.

## Background

Following the completion of several whole genome sequencing projects a considerable effort has been focused on genome-wise functional analyses of a number of organisms (reviewed in [1]). Some of the most popular methods are the study of gene expression by microarrays and phenotypic analyses from gene knock-downs by means of RNA interference techniques [2,3]. The success of these methods relies in the ability of reagent oligonucleotides to specifically recognise single species of transcripts within the complex mixture present in the studied cells. Therefore, when designing probes, primers and siRNAs, the sequence specificity of candidate oligonucleotides must be assessed in order to minimise potential cross-hybridisations and off-target effects [4,5]. Although cross-reaction events have been described between siRNAs and molecules of limited sequence similarity [6,7], the determination of specificity routinely requires the identification of oligonucleotides that are identical in sequence only to the intended target. This uniqueness assessment is usually calculated every time that a new reagent needs to be designed. However, given the availability of complete genome sequences for a number of organisms, all their unique regions could be calculated, stored and made publicly available, for example, via an online resource. In addition, this resource could also take advantage of the known contextual relationships between transcripts within a gene to categorise uniqueness at the gene and transcript levels to, for example, easily discriminate between unique regions shared by all transcripts and those exclusive to individual alternative splicing variants. This information would simplify the process of oligo design by abolishing the step to determine unique fragments, with the desired range of action, within the gene/transcript of interest. At present, no such resource is available.

The [X]uniqMAP database has been developed to store and present already pre-calculated unique regions for all EnsEMBL transcripts of the human and mouse genomes [8], the most widely used systems for the study of mammalian genetics. It also records those unique fragments that are shared between them, which could help to identify sequences to simultaneously target equivalent genes between these two organisms. [X]uniqMAP differs from standard genomic browsers in that it uses genome comparisons to reveal unique regions within and between organisms. These regions are presented at both gene and transcript levels. The data stored in [X]uniqMAP can be retrieved via a user-friendly web interface or as downloadable FASTA files, and it should be useful for small- as well as large-scale projects for which the identification of unique DNA regions is required.

## Construction and content

[X]uniqMAP is a collection of three databases: human and mouse uniqMAP as well as XuniqMAP. The first two collect the unique DNA 19-mers for all gene/transcripts within the two genomes. XuniqMAP collects those unique fragments within human and mouse that are also shared between them. The determination of the unique regions within a genome comprises three steps: (i) the construction of a set of non-redundant (NR) sequences, keeping track of the gene structure, from all the transcripts of each gene; (ii) self-comparison of the NR sequence set and storage of those positions found to be redundant; and (iii) deduction of the unique regions from regions identified in (ii) and gene structure data from (i).

**Figure 1:**
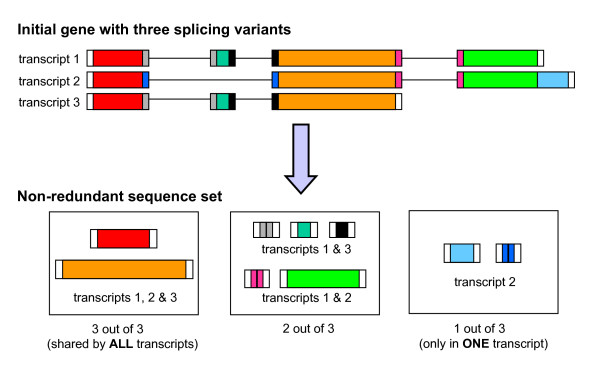
Building the non-redundant sequence set. The schema depicts an example for the establishment of the NR sequence set for a gene with three splicing variants. The different fragments are grouped according to their presence across all transcripts as described in the main text. Notice that these fragments (coloured) comprise only the central positions of all possible 19-mers and therefore transcript ends are not included (blank boxes at the top of the Figure). However, in the final NR sequence set (bottom) the 5' and 3' ends will be added to their corresponding fragments and the ends of the other fragments will be extended until they account for the full-length sequences of all 19-mers they represent. The philosophy behind this procedure is similar to that previously described by others [4].

For each genome, the NR sequence set was established as follows. First, the central positions (i.e. the tenth nucleotide) for all possible 19-mers of all EnsEMBL transcripts, including non-coding RNAs and pseudogenes, were calculated. This length is around the standard minimum length for oligo design. All the central positions that fell within nine bases from the exonic boundaries were considered to be part of the regions that join exons whereas the others were part of the exon body (Figure 1, top). Then, single copies of exonic ends, combined as seen in all transcripts, and exon bodies were extracted from each gene, storing in the database the genomic coordinates for these fragments as well as information about the number of transcripts they came from (Figure 1, bottom). In the final NR sequence set, the fragments were extended by nine nucleotides at both ends to account for the full-length 19-mers they represented (Figure 1, bottom). Non-coding RNAs and pseudogenes were included to ensure that as many transcripts that could potentially be found in the cell were considered, since we reasoned that it would be better to underestimate the number of unique regions that to accept spurious ones due to the underestimation of the amount of transcriptable DNA.

The next step was to compare the sequences of the NR set with each other in order to determine which 19-mers were shared between several genes or transcripts. Prior to this, repeats and low complexity regions were filtered out [9] and the sequences were fragmented into all possible overlapping 19-mers excluding those containing filtered positions. Afterwards, the 19-mers were compared against the original, unfiltered, NR set using the exonerate program [10], recording only those matches, in either sense, at least 18 bases long and sharing 18 identities. At the end, the genomic coordinates of the central positions of the 19-mers containing filtered positions or matching to others were stored.

Finally, the unique regions were calculated from the above values kept in the database as follows. For regions unique to a gene, the segments shared by all transcripts of that gene were located onto the exonic regions from which the filtered and redundant positions were subsequently subtracted, yielding the remaining positions as unique. An identical procedure was applied to uncover the unique regions in individual transcripts with the exception that the positions initially mapped onto the transcripts were those exclusive to them. This discrimination of gene/transcript levels in unique regions is important for the design of oligos with the desired range of action. For human, unique regions were identified in 26704 out of 31274 genes, and 23438 transcripts from a total of 36161 transcripts from genes with annotated alternative splicing variants. For mouse, 26135 (out of 27735) genes and 11350 (out of 11807) transcripts contained unique regions.

For the calculation of unique positions shared between genomes, the two sets of unique fragments within the human and mouse genomes were compared against each other, storing information only for pairs of genes that shared at least five identical 19-mers in either sense. This arbitrary cut-off resulted almost completely in one-to-one, typically orthologous, relationships between human and mouse genes. The final set contains 15104 pairs made from 14482 and 14645 genes from human and mouse, respectively. As above, the gene/transcript relationships from each organism were transferred to the shared sequences which were then labelled according to their potential range of action across both organisms (see also next section for further details).

**Figure 2:**
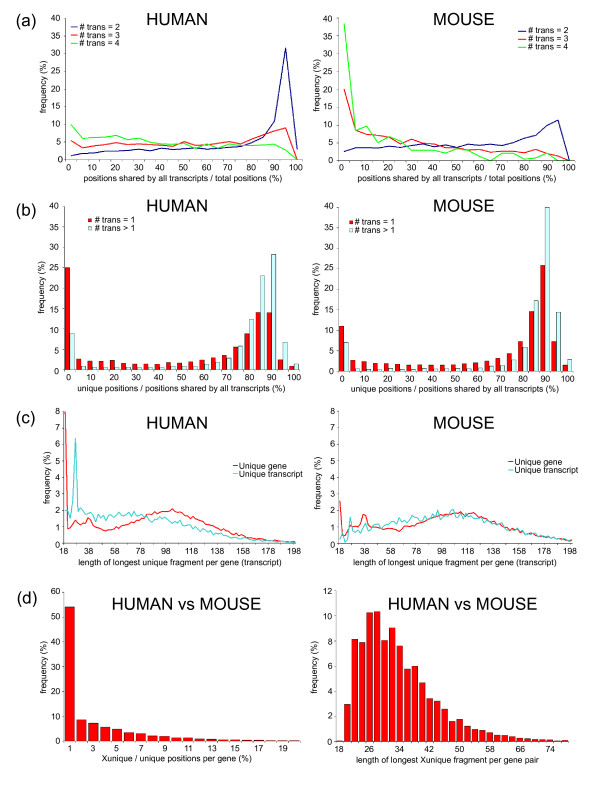
[X]uniqMAP statistics based on EnsEMBL version 40. Figures (a) to (c) summarise the data for the intra-comparisons within the human (left) and mouse (right) genomes. **(a) **The plots with the distributions of the proportion of exonic regions shared by all transcripts within a gene indicate that for some genes with high number of splicing variants it may be impossible to find a region to target simultaneously all their transcripts. **(b) **Distributions of the proportion of unique 19-mers found for genes, excluding pseudogenes, with single (red) and multiple transcripts (cyan) show that most genes present a high degree of uniqueness, although for nearly 25% of human genes the level of uniqueness is poor, i.e. between 0 and 5%. **(c) **Graphs summarising the lengths of the longest unique fragments found for each gene (red) or individual transcripts (blue). **(d) **Statistics from the inter-species comparisons of the human and mouse unique regions. The histograms correspond to the proportion of unique positions shared between the two organisms with respect to the total number of unique positions within each one of them (left) and the distribution of the longest unique fragments shared between the gene pairs (right).

A summary of the information held currently in the database, based on EnsEMBL release 40, can be found in Figure 2. Figure 2a shows that with an increasing number of transcripts per gene it can sometimes be difficult to find unique regions at the gene level since the proportion of overlapping regions between all transcripts decreases, in some cases totally. In the latter, no single oligo would be able to act at the gene level since it would never recognise all their possible transcripts. Nevertheless, when regions shared by all transcripts from a gene exist, 85% and 94% of human and mouse genes, respectively, do present unique regions that, in more than half of the cases, extend to 60% (human) and 72% (mouse) of their lengths (Figure 2b). The distribution of the maximum lengths of the unique regions per gene/transcript shows that it is possible to find unique fragments that are at least 40 bases long in 80% (human) and 86% (mouse) of the cases (Figure 2c). From the comparison of unique regions across the genomes, we observed that 15104 human-mouse gene pairs share identical unique regions, although these fragments only represent a small subset of the total length of the intra-genomic unique regions (Figure 2d, left) and in 76% of the cases the longest fragments are shorter than 40 bases (Figure 2d, right).

## Utility and discussion

[X]uniqMAP contains a collection of the unique regions of all gene/transcripts in human and mouse as well as those unique sequences shared between their orthologs. For high throughput studies the complete data sets can be downloaded as FASTA files from the web site for further in-house calculations. The description lines of the sequences in the FASTA files contain all the appropriate information about the genomic co-ordinates and gene/transcript coverage of the fragments (see below for a more detailed explanation). On the other hand, in the more general case of experimentalists interested in only one or just few genes, this information can be retrieved interactively from the web site by searching the database either with an identifier or a nucleotide sequence.

When a sequence is provided, this will be BLASTed [11] against the set of unique sequences stored in the database. Only nucleotide sequences are allowed as queries. The search parameters have been adjusted to effectively deal with queries of any length, reporting only those local matches that are highly similar to the query, at least 19 nuleotides long and contain no gaps. The results page of the sequence search comprises graphical and sequence representations of the regions of the oligonucleotide query matching to those in the database highlighting the type of unique sequences, i.e. gene versus transcript, matched as well as the presence of mismatches if any.

**Figure 3:**
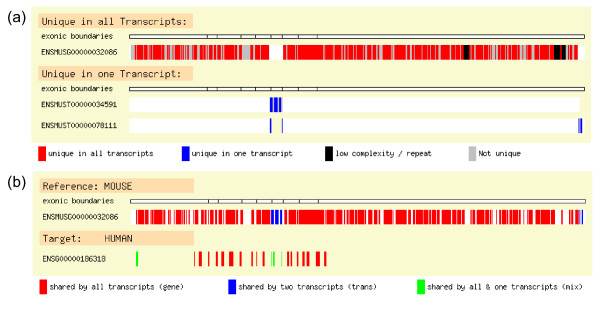
Graphical representation of unique genomic regions. **(a) **The graphical display for the unique regions within a genome depicts the full-length sequences, without common introns, as white rectangles with the unique regions in red for genes (top) and blue for individual transcripts (bottom). Repeats or low complexity regions are highlighted in black and those redundant in grey. **(b) **For the unique regions shared across genomes, the two levels of the display correspond to all the unique regions of the gene in the reference organism and those matched by the target organism. The colour-coded scheme for the reference is the same as in (a). For the target, the shared unique positions are placed relative to those matched with the reference and the colours represent all the possible combinations that can be found between the shared sequences, as explained in the main text.

Only gene and transcript Ensembl identifiers are valid when searching by an identifier. Once the identifier is provided, the database will recognise automatically to which organism it corresponds, therefore the organism option does not need to be specified unless to target directly the set of mouse-human shared unique regions. For each gene entry a web page will show all the information stored in the database by means of graphical and sequence displays. In uniqMAP, the graphical display is split into two levels, namely gene and transcript, corresponding to regions shared by all transcripts or by a single transcript, respectively (Figure 3a). In both representations, the full-length sequences are depicted as white rectangles where the introns common to all transcripts have been removed for clarity but the relative coordinates of exon boundaries are still consistently preserved between the transcripts. An approximate representation of the boundaries for the longest combined exons of the gene is shown at the top of each level. At both levels, the regions presenting repeats or low complexity are highlighted in black whereas those matching other potential transcriptable fragments within the genome are coloured in grey. Unique regions are shown in red for genes (Figure 3a, top) and blue for individual transcripts (Figure 3a, bottom). The sequence display is organised in a similar way, using the same colour-coded scheme as for the graphical display. In either case, only the central positions (see Construction and Content section) of the unique 19-mers are indicated, and because of this, nine neighbouring bases should be included at each end in order to obtain the desired 19-mer. Alternatively, the sequences for all the unique fragments, ordered by decreasing length, can automatically be retrieved via the provided links. In this case, the description line for each fragment contains the following information:

>gene_id|transcript_id|symbol|chromosome_number:genomic_coordinates(strand)|fragment_length

where the value of symbol can be either Ug or Ut, depending on whether the fragment is shared by all transcripts in a gene or unique to a single transcript, respectively. For the former case, gene_id and transcript_id will be the same and it will correspond to the EnsEMBL gene identifier.

In XuniqMAP, the display is also split into two levels, namely reference and target, corresponding to all the unique regions of the gene in the reference organism and those matched by the target organism, respectively (Figure 3b). The colour-coded scheme for the reference is the same as in uniqMAP, i.e. red for unique regions shared by all transcripts and blue for those specific to individual transcripts (Figure 3b, top). For the target, the shared unique positions are placed relative to those matched with the reference and the colours represent all the possible combination of matches that can be found between the shared sequences: (i) red when they are present in all transcripts for both genes; (ii) blue if present only in individual transcripts for both genes; and (iii) green when present in all the transcripts of one gene but only in a single transcript of the other (Figure 3b, bottom). In the sequence display, only the gene of the target sequence is shown where the central positions are coloured as in the graphical display. Links are also provided for the retrieval of the sequences in FASTA format, for each type of match. In this case, the description lines for the fragment contain the following information:

>target_gene_id:target_transcript_id:target_symbol|reference_gene_id:reference_transcript_id:reference_symbol|target_chromosome_number:targtet_genomic_coordinates(target_strand)|fragment_length

where, as in uniqMAP, the symbol nomenclature will describe whether the fragment is shared or not by all transcripts.

## Conclusion

[X]uniqMAP is a collection of databases that stores all unique regions within human and mouse gene/transcripts as well as the sub-set of these unique regions that are identical between these two organisms. All the information is provided in the adequate genomic context for each gene, reflecting the relationships between their transcripts. This discrimination between regions shared by all transcripts of a gene from those exclusive to individual transcripts should be useful to determine the range of action desired when designing oligos. For oligo design, [X]uniqMAP information could be fed, if necessary, directly to downstream pipelines (i) to assess further the adequacy of some segments as, for example, RNAi or primers; or (ii) for extra filtering if lower similarity to other potential cross-reactive molecules as well as the precise location of the mismatched positions must be accounted for. [X]uniqMAP is based on EnsEMBL and is updated in parallel with EnsEMBL to reflect changes in genome assemblies or gene builds.

## Availability and requirements

[X]uniqMAP is available online from . Enquiries on technical or data access issues can be mailed to webmaster@mitocheck.org.

## Authors' contributions

RD conceived the initial idea about a database to store unique genomic regions. JLJ designed and implemented the data pipeline and database. JLJ and RD analysed the data and wrote the paper. Both authors read and approved the final manuscript.

## References

[B1] Lee NH (2005). Genomic approaches for reconstructing gene networks. Pharmacogenomics.

[B2] Eisen MB, Brown PO (1999). DNA arrays for analysis of gene expression. Methods Enzymol.

[B3] Vanhecke D, Janitz M (2004). High-throughput gene silencing using cell arrays. Oncogene.

[B4] Naito Y, Yamada T, Ui-Tei K, Morishita S, Saigo K (2004). siDirect: highly effective, target-specific siRNA design software for mammalian RNA interference. Nucleic Acids Res.

[B5] Nielsen HB, Wernersson R, Knudsen S (2003). Design of oligonucleotides for microarrays and perspectives for design of multi-transcriptome arrays. Nucleic Acids Res.

[B6] Jackson AL, Bartz SR, Schelter J, Kobayashi SV, Burchard J, Mao M, Li B, Cavet G, Linsley PS (2003). Expression profiling reveals off-target gene regulation by RNAi. Nat Biotechnol.

[B7] Martinez J, Tuschl T (2004). RISC is a 5' phosphomonoester-producing RNA endonuclease. Genes Dev.

[B8] Birney E, Andrews D, Caccamo M, Chen Y, Clarke L, Coates G, Cox T, Cunningham F, Curwen V, Cutts T (2006). Ensembl 2006. Nucleic Acids Res.

[B9] Smith AFA, Green P (1996). RepeatMasker. http://repeatmasker.genome.washington.edu.

[B10] Slater GS, Birney E (2005). Automated generation of heuristics for biological sequence comparison. BMC Bioinformatics.

[B11] Altschul SF, Gish W, Miller W, Myers EW, Lipman DJ (1990). Basic local alignment search tool. J Mol Biol.

